# Phytoplankton Cell Size: Intra- and Interspecific Effects of Warming and Grazing

**DOI:** 10.1371/journal.pone.0049632

**Published:** 2012-11-30

**Authors:** Kalista Higini Peter, Ulrich Sommer

**Affiliations:** Helmholtz Centre for Ocean Research Kiel (GEOMAR), Kiel, Germany; University of Hamburg, Germany

## Abstract

Decreasing body size has been suggested as the third universal biological response to global warming after latitudinal/altitudinal range shifts and shifts in phenology. Size shifts in a community can be the composite result of intraspecific size shifts and of shifts between differently sized species. Metabolic explanations for the size shifts dominate in the literature but top down effects, i.e. intensified size-selective consumption at higher temperatures, have been proposed as alternative explanation. Therefore, we performed phytoplankton experiments with a factorial combination of warming and consumer type (protist feeding mainly on small algae vs. copepods mainly feeding on large algae). Natural phytoplankton was exposed to 3 (1^st^ experiment) or 4 (2^nd^ experiment) temperature levels and 3 (1^st^ experiment: nano-, microzooplankton, copepods) or 2 (2^nd^ experiment: microzooplankton, copepods) types of consumers. Size shifts of individual phytoplankton species and community mean size were analyzed. Both, mean cell size of most of the individual species and mean community cell size decreased with temperature under all grazing regimes. Grazing by copepods caused an additional reduction in cell size. Our results reject the hypothesis, that intensified size selective consumption at higher temperature would be the dominant explanation of decreasing body size. In this case, the size reduction would have taken place only in the copepod treatments but not in the treatments with protist grazing (nano- and microzooplankton).

## Introduction

Changed biogeographic distributions and seasonal patterns are the two most general and most often reported biological responses to global climate warming [1], [2], [3]. Recently, a debate emerged whether a reduction in body size can be considered the third universal response to warming [4], [5]. Such a trend would conform to classic biogeographic rules, Bergmann's rule [6] and James' rule [7] which predict smaller body sizes in warmer climates. While those rules were initially coined for endotherms and explained by easier thermoregulation at lower surface:volume ratios, they were later extended to ectotherms. A physiological explanation was provided by the Temperature Size Rule (TSR) which predicts a smaller final body size at maturity because maturation is accelerated more strongly by warming than somatic growth [8], [9]. Changed body size distributions in a community or a trophic level consist of three different components: species replacements, changes in age structure of individual populations and size changes at a defined age or developmental stage within species [10].

While size reduction in response to warming seems to become an accepted rule in spite of counter-examples (Table 1 in [4] for vertebrates; [11] for phytoplankton) there is no consensus about the underlying causality, given that the prevailing explanations are not being mutually exclusive. Explanations under the roof of the TSR [8] explicitly refer to size shifts within species. Community or trophic level wide shifts brought about by dominance shifts between species are often explained by intensified resource competition under higher temperatures and competitive advantages for smaller species [12], [13], [14], [15], [16], [17]. As an alternative explanation, intensified predation at higher temperatures has been suggested, particularly for primary producers, because heterotrophic metabolic rates grow faster with temperature than photosynthesis [17], [18], [19], [20]. The predation effect should be particularly strong when predators prefer larger prey, such as copepods as predators on phytoplankton [21], [22]. However, the predation effect should be reversed or partially reversed, if the prevailing predators prefer small prey. In this case, stronger predation at higher temperature would lead to a stronger removal of small prey.

**Table 1:** Higher taxon and mean cell volume (*V_m_*; µm^3^; grand mean across all treatments) of phytoplankton species, arranged in descending order of size.

Species	taxon	*V_m_*
**experiment 1**		
*Scrippsiella trochoidea*	Dinophyta	1046
*Dictyocha speculum*	Dictyochophyceae	235
*Teleaulax amphioxeia*	Cryptophyta	191
*Chaetoceros similis*	Bacillariophyceae	88.7
Picophytoplankton	diverse higher taxa	5.55
**experiment 2**		
*Ditylum brightwellii*	Bacillariophyceae	12627
*Guinardia delicatula*	Bacillariophyceae	2207
*Amphidinium* sp.	Dinophyta	987
*Chattonella* sp.	Raphidophyceae	968
*Chaetoceros brevis*	Bacillariophyceae	960
*Teleaulax amphioxeia*	Cryptophyta	144
*Skeletonema* cf. *costatum*	Bacillariophyceae	93.7
*Chaetoceros gracilis*	Bacillariophyceae	51.8
Picophytoplankton	diverse higher taxa	4.62

In order to test the role of predation in temperature-size relationships we chose marine phytoplankton as a model system because of (a) their importance as primary producers by contributing ca. 50% of global primary production, (b) their short generation time and ease of experimental handling, and (c) because the size effects of their main predators are well known. Copepods tend to suppress medium to moderately large sized phytoplankton (lower limit 10^2^ to 10^3^ µm^3^, upper limit 10^4^ or 10^5^ µm^3^ cell volume, [22]) but also microzooplankton (mainly ciliates and heterotrophic dinoflagellates). Thereby, they release smaller phytoplankton from grazing pressure, because most microzooplankton feed on phytoplankton <500 to 1000 µm^3^ [23]. Overall, interspecific grazer effects should have a stronger impact on community mean body size than intraspecific ones, because intraspecific size differences are usually much smaller than interspecific ones.

Our working hypotheses were:

1. Cell size of individual phytoplankton species decreases with temperature.
2. Temperature effects on cell sizes of species will be modified by grazers.
3. Warming leads to a decrease of community mean cell size of phytoplankton.
4. Temperature effects on community mean cell sizes will be modified by grazers.
  4a (strong version): There will be a reversal of sign in the temperature – size relationship (negative under copepod grazing, positive under protozoan grazing)
  4b (weak version): Different grazer guilds will only modify the response, but not reverse it.

## Materials and Methods

### Experiment Design

The first experiment was conducted from 1^st^ to 28^th^ April 2011. The experiment was performed in Erlenmeyer flasks of 700 mL incubated in temperature and light controlled climate cabinets. Twenty seven Erlenmeyer flasks of 700 mls were filled with natural seawater from 1 to 3 m depth from Kiel Fjord (Western Baltic Sea) which contained the natural spring plankton community. They were placed in 3 climate cabinets with temperatures of 4.5, 6.5, and 10.5°C, respectively. We used three grazing treatments, N: nanozooplankton only (natural seawater sieved through a 20 µm gauze), M: micro- and nanozooplankton (natural seawater sieved through a 200 µm gauze), and C: nano-, microzooplankton and copepod (natural seawater sieved through a 200 µm gauze and supplemented with the copepod *Acartia tonsa* nauplii at an initial density of 10 ind. L^−1^ after one week). Thus, the treatments N, M and C represented a gradient in grazer size. The three temperature levels (4.5, 6.5 and 10.5°C) were combined with the three grazing regimes in a full factorial design, resulting in 9 treatment combinations; each treatment replicated 3×.The coldest temperature (4.5°C) corresponded to the ambient water temperature in the Kiel Fjord at the time of sampling. The light intensity was 293 µmol m^−2^ s^−1^ and the light:dark cycle 13:11 hrs, in accordance with the season of the experiment. Erlenmeyer flasks were mixed by shaking twice per day. The salinity was 15.6 PSU. The water received no nutrient addition. Initial concentrations were 7.34 µmol l^−1^ nitrate, 2.6 µmol l^−1^ ammonium, 0.13 µmol l^−1^ dissolved phosphate, and 16 µmol l^−1^ dissolved silicate.

The second experiment was conducted from 5^th^ to 28^th^ July 2012. We used twenty four indoor mesocosms of 300 L filled with natural summer plankton communities direct pumped from Kiel Fjord, western Baltic Sea. Copepods were excluded by sieving. The two grazing treatments consisted of absence of copepods (M) and of the addition of freshly caught copepods (C) at an initial densitiy of 15 ind L^−1^. Copepods were caught with a 200 µm plankton net with a cod end and evenly distributed to the C-mesocosms. The natural community was strongly dominated (>95%) by *Acartia tonsa* which made it easy to offer the same species composition to all mesocosm. The four temperature levels (8, 12.5, 15.5 & 18°C) were combined with the two grazing regimes in a fully factorial design, resulting into 8 treatment combination each replicated 3×. The coldest temperature corresponded to the ambient water temperature in the Kiel Fjord at the time of sampling. The light intensity was 249 µmol m^−2^ s^−1^ and the light : dark cycle 14:10 hrs. Because of low in situ nutrient concentration, nutrients were supplemented with moderate additions of nitrate and phosphate, leading to starting concentrations of 10.6 µmol l^−1^, 0.6 µmol l^−1^ NH_4_, 0.8 µmol l^−1^ PO_4_, and 7.0 µmol l^−1^ dissolved Si. Mixing was by done manually by using standard boat paddle three times per day at 7.30 am, 2 pm & 8 pm. No specific permits were required for the described field samplings. The sampling site is not privately owned or protected in any way and no endangered species have been sampled.

### Sampling and analysis

Samples for phytoplankton counts were taken once per week and immediately fixed with Lugol's iodine. Mixing was done before sampling to insure homogeneity. Water temperature, fluorescence, salinity and pH were measured every day to monitor the system. Phytoplankton smaller than 5 µm were measured and sized by flow cytometry (FACScalibur, Becton Dickinson). Flow cytometry samples were sampled and immediately fixed with formeldehyde at 2% final concentration in vials. The vials were sealed and stored at −80°C until analysis. In addition, these algae were identified by using a scanning electron microscope (SEM). SEM samples were taken and immediately filtered by using Nuclepore Track-Etch Membrane (Whatman) and dried at 60°C for 60 minutes. Only the diatom *Chaetoceros gracilis* could be identified, while the preparation method permitted no identification of picoplankton. Cell volumes of picoplankton were calculated as volumes of sphere.

Phytoplankton bigger than 5 µm were counted using the inverted microscope method [24] with settling cylinders of 50 ml and composite chambers with a bottom area of 500 mm^2^. Cells were allowed to settle for 24 h before counting. It was attempted to count at least 100 cells of each taxon to achieve 95% confidence limits of ±20%. Cell size measurements were performed with the samples from the end of the experiments in order to get maximum time for the treatment to take effect. This was a period of slowly declining biomass after an interim peak in all treatments of both experiments. Linear cell dimensions were measured with the AxioVision programme (Zeiss) and the cell volumes were calculated after approximation to geometric models [25]. Twenty randomly selected cells from each species per sample were measured. Species biomass (*B_i_*) was calculated form specific abundances (*N_i_*) and cell volumes (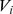): B_i = _N_i_*V_i_. Community mean cell size (*V_c_*) was calculated by dividing the total biomass by the total cell number:
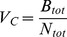



Dissolved nutrients were measured according to oceanographic standard methods. At the end of experiment 2 also particulate matter C and N content were measured with a CHN analyzer (Fisons, 1500 N, Fisons Instruments, MA, USA).

### Statistical analysis

The significance of temperature and grazing effects and their interaction was tested by ANOVA (STATISTICA 7). The quantitative relationship between size and temperature was analyzed by regressions of cell sizes and biomass on temperature conducted separately for each grazing treatment. The best fits were obtained after logarithmic transformation of both the dependent and the independent variable.

## Results

### Cell volumes of individual species

A total of 11 microsocpically counted species was abundant enough to perform size measurements, four species from experiment 1, the silicoflagellate *Dictyocha speculum*, the dinoflagellate *Scrippsiella trochoidea*, the cryptophyte *Teleaulax amphioxeia*, and the diatoms *Chaeotoceros similis*, *and* seven species from experiment 2, the dinoflagellate *Amphidinium* sp., the diatoms *Guinardia delicatula*, *Chaetoceros brevis*, *Chaetoceros gracilis, Ditylum brightwellii, Skeletonema* cf. *costatum* , the cryptophyte *Teleaulax amphioxeia* and the raphidophyte *Chattonella* sp. (Table 1). Picophytoplankton counted by flow cytometry were treated as a collective category without species distinction. Three species disappeared in the warmer treatments, *C. similis* at 10.5°C in experiment 1, *C. brevis* and *D. brightwelii* at 15.5 and 18.5°C in experiment 2.

**Figure 1:**
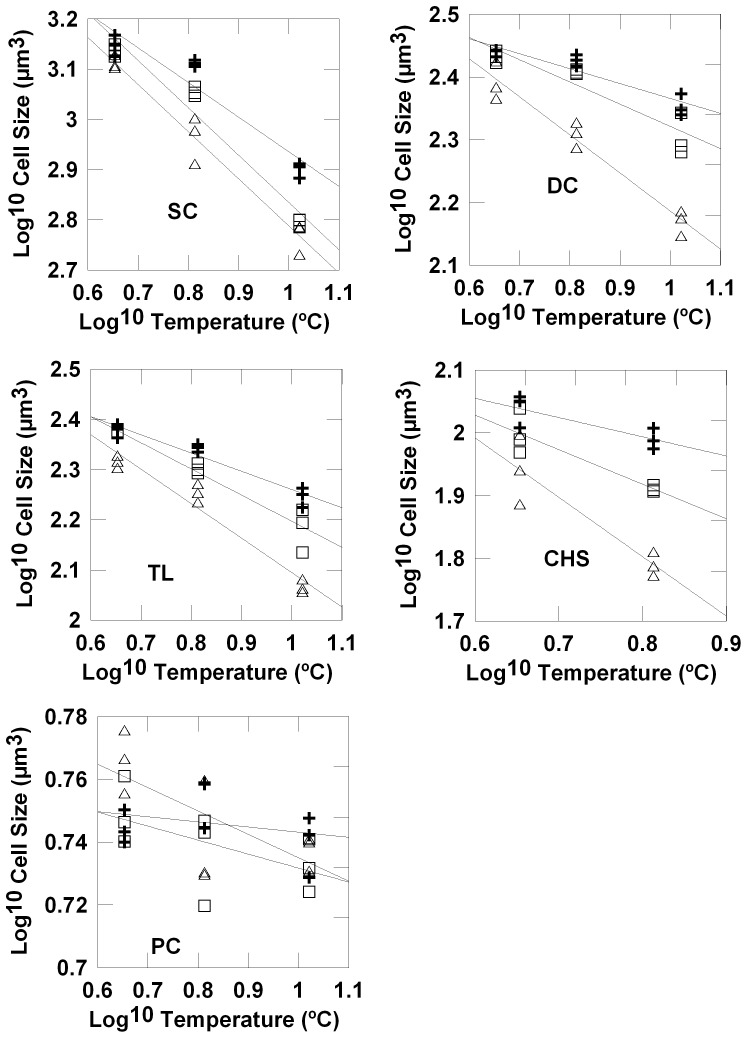
Temperature and grazing effects on the size of individual phytyplankton species, experiment 1. Regressions of mean cell sizes of individual species (log^10^ transformed, µm^3^) on temperature (log^10^ transformed, °C) for the different grazing regimes (nanozooplankton-N: crosses; microzooplankton-M: open squares; copepods-C: open triangles; SC: *Scrippsiella trochiodea*, DC: *Dictyocha speculum*, TL: *Teleaulax amphioxeia*, CHS: *Chaetoceros similis*, PC: picophytoplankton.

**Figure 2:**
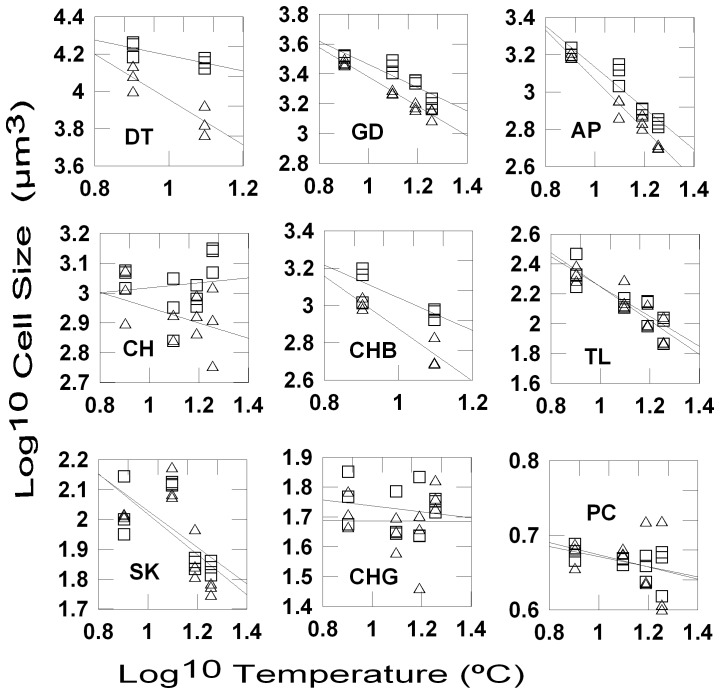
Temperature and grazing effects on the size of individual phytyplankton species, experiment 2. Regressions of mean cell sizes of individual species (log^10^ transformed, µm^3^) on temperature (log^10^ transformed, °C) for the different grazing regimes (microzooplankton-M: open squares; copepods-C: open triangles); DT: *Ditylum brightwellii*, GD: *Guinaridia delicatula*, AP: *Amphidinium* sp., CH: *Chattonella* sp., CHB: *Chaetoceros brevis*, TL: *Teleaulax amphioxeia*, SK: *Skeletonema* cf. *costatum*, CHL: *Chaetoceros gracilis*; PC: picophytoplankton.

The majority of species species (*D. speculum, S. trochoidea, T. amphioxeia, C. similis*, and picophytoplankton in experiment 1; *G. delicatula, A.* sp., *T. amphioxeia, C. brevis, D. brightwelii*, and *S.* cf. *costatum* in experiment 2) decreased in cell size with increasing temperature (Fig. 1 & 2; Table 2) while there was no significant temperature effect for *C. brevis* (experiment 2), *C. gracilis* (experiment 2) and for picophytoplankton in experiment 2. . The grazing effect was significant in all cases except for *C. gracilis* (experiment 2), *S.* cf. *costatum* (experiment 2), *T. amphioxeia* (experiment 2), and picophytoplankton (experiments 1 and 2). Significant temperature – grazing interaction were found in most species during experiment 1 (*D. speculum, S. trochoidea, T. amphioxeia, C. similis*) and 4 species during experiment 2 (*G. delicatula, A.* sp., *C. brevis*, *D. brightwellii*). The mean cell sizes of all species showing a significant response to grazing declined with grazer size, i.e. at a given temperature cell sizes were smallest in the C-treatments. The grazing influence on the slopes of the size-temperature regressions showed interspecific differences. The slope was either most strongly negative in the C-treatments or there were no differences in the slope (*C. gracilis, S.* cf. *costatum, S. trochoidea* and picophytoplankton (Table 3, Fig. 1 & 2).

**Table 2:** Two-factor ANOVA of temperature and grazing effects on cell sizes.

Species	p-temperature	p-grazing	p-interaction	R^2^
**experiment 1**				
*S. trochoidea*	<0.001	<0.001	0.06	0.86
*D. speculum*	<0.001	<0.001	0.002	0.77
*T. amphioxeia*	<0.001	<0.001	0.0001	0.83
*C. similis*	<0.001	<0.001	0.04	0.77
Picophytoplankton	0.01	0.23	0.10	0.37
**experiment 2**				
*D. brightwellii*	<0.001	<0.001	0.007	0.89
*G. delicatula*	<0.001	<0.001	<0.001	0.85
*A*. sp.	<0.001	<0.001	0.04	0.82
*Chattonella* sp.	0.07	0.04	0.21	0.34
*C. brevis*	<0.001	0.005	0.003	0.83
*T. amphioxeia*	<0.001	0.65	0.73	0.72
*S*. cf. *costatum*	<0.001	0.39	0.5	0.81
*C. gracilis*	0.9	0.25	0.63	0.12
Picophytoplankton	0.47	0.94	0.91	0.16

**Table 3:** Regressions (model: y = ax+b) of log^10^ cell volume (µm^3^) on log^10^ temperature (°C) for the different species and grazing regimes.

Species	Grazing	a	b	p	R^2^
**Experiment 1**					
*S. trochoidea*	N	−0.69	3.6251	0.0001	0.79
	M	−0.9399	3.7702	0.0004	0.85
	C	−0.9655	3.7452	0.00007	0.87
*D. speculum*	N	−0.2378	2.6034	0.0004	0.78
	M	−0.3447	2.6685	2.6685	0.82
	C	−0.5951	2.7888	2.7888	0.85
*T. amphioxeia*	N	−0.3631	2.6234	0.0004	0.83
	M	−0.4627	2.6552	0.002	0.76
	C	−0.6863	2.7812	0.0001	0.86
*C. similis*	N	−0.3087	2.2362	0.05	0.64
	M	−0.5565	2.3580	0.014	0.79
	C	−0.9590	2.5629	0.011	0.82
Picophytoplankton	N	−0.0164	0.7596	0.46	0.07
	M	−0.0446	0.7764	0.11	0.32
	C	−0.0558	0.7962	0.15	0.26
**Experiment 2**					
*D. brightwellii*	M	−0.5876	4.7962	0.0036	0.87
	C	−1.5577	5.4801	0.0035	0.85
*G. delicatula*	M	−0.7800	4.2430	<0.001	0.77
	C	−0.9885	4.3677	<0.001	0.87
*A*. sp.	M	−1.1708	4.3032	<0.001	0.78
	C	−1.4190	4.5027	<0.001	0.87
*Chattonella* sp.	M	+0.0830	2.9345	0.6805	0.0177
	C	−0.2566	3.2076	0.2068	0.1541
*C. brevis*	M	−0.8787	3.9191	0.0441	0.67
	C	−1.4088	4.2833	0.0055	0.88
*T. amphioxeia*	M	−1.0012	3.2500	0.00020	0.75
	C	−1.1354	3.3832	0.00007	0.80
*S*. cf. *costatum*	M	−0.612	2.641	0.0244	0.41
	C	−0.6757	2.6933	0.0214	0.43
*C. gracilis*	M	−0.1004	1.8374	0.5758	0.0324
	C	−0.0036	1.6902	0.9871	0.0024
Picophytoplankton	M	−0.0972	0.7742	0.0429	0.34
	C	−0.051	0.7244	0.4640	0.06

### Total phytoplankton biomass and mean cell size

**Figure 3:**
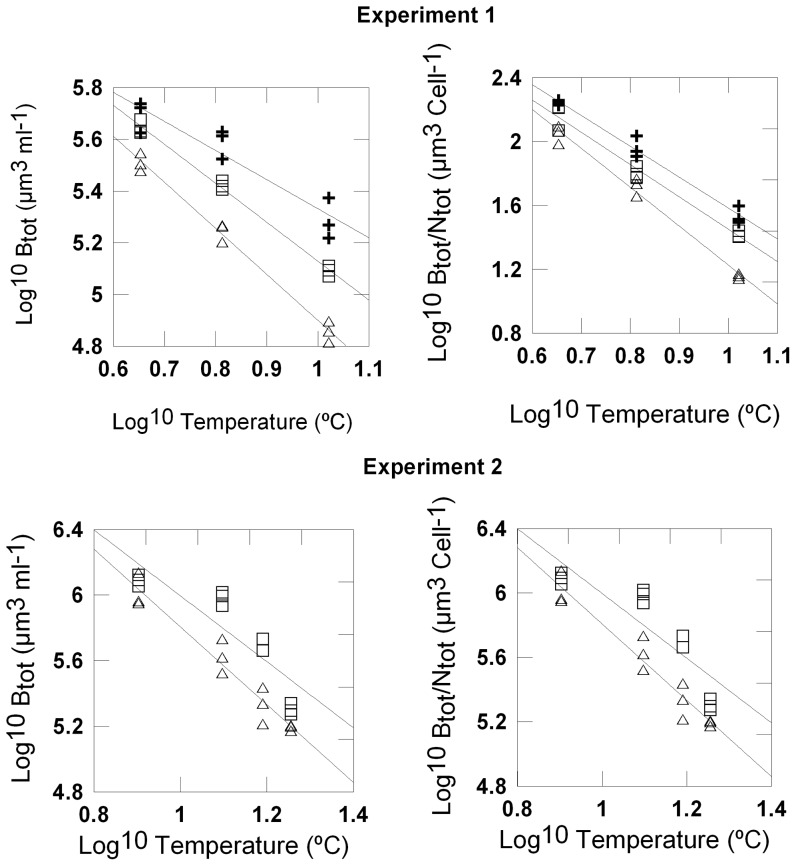
Temperature and grazing effects on biomass and mean size of the phytoplankton community. Regressions of total biomass (B_tot_) and community cell sizes (B_tot_/N_tot_) to temperature (log^10^ transformed, °C) for the different grazing regimes (nanozooplankton-N: crosses; microzooplankton-M: open squares; copepods-C: open triangles).

Total phytoplankton biomass and community mean cell size declined with temperature and in the direction of N – M - C. The temperature and grazing effects and their interaction on total biomass and on mean cell size were significant in both experiments (Table 4, Fig. 3). The slopes of the biomass-temperature and of the size-temperature regressions became more negative with increasing grazer size (Table 5).

**Table 4:** Two-factor ANOVA of temperature and grazing effects on total Biomass (B_tot_) and community cell size (B_tot_/N_tot_).

Experiment 1				
	p-temperature	p-grazing	p-interaction	R^2^
*B_tot_*	<0.001	<0.001	0.004	0.86
*B_tot_/N_tot_*	<0.001	<0.001	0.02	0.75

Experiment 2				
*B_tot_*	<0.001	<0.001	<0.001	0.86
*B_tot_/N_tot_*	<0.001	<0.001	0.003	0.87

**Table 5:** Regressions (model: y = ax+b) of log^10^ total biomass (B_tot_) and Community cell size (B_tot_/Nt_ot_) on log^10^ temperature (°C) for the different species and grazing regimes.

	Grazing	a	b	p	R^2^
**Experiment 1**					
B_tot_	N	−1.1296	6.4594	0.002	0.77
	M	−1.4114	6.5359	0.0001	0.84
	C	−1.7091	6.629	0.00003	0.86
B_tot_/Nt_ot_	N	−1.9371	3.5187	0.00004	0.80
	M	−2.1193	3.5417	0.00001	0.86
	C	−2.4582	3.678	0.000002;	0.82
**Experiment 2**					
B_tot_	M	−2.066	8.0606	0.0002	0.75
	C	−2.4534	8.2585	<0.0001	0.84
B_tot_/Nt_ot_	M	−2.2879	4.6646	0.0007	0.70
	C	−2.9993	5.1787	0.00002	0.84

### Taxonomic composition

**Figure 4:**
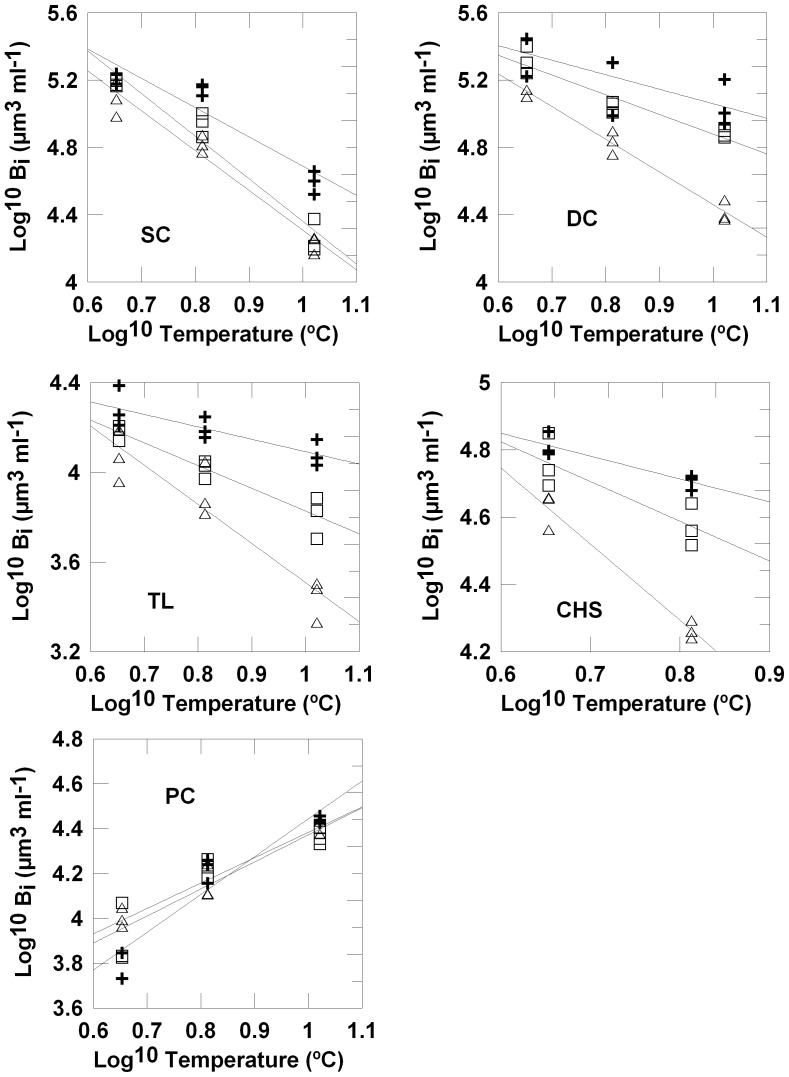
Temperature and grazing effects on the biomass of individual phytoplankton species, experiment 1. Regressions species specific biomass (log^10^ transformed, µm^3^ml^−1^) on temperature (log^10^ transformed, °C) and grazing regimes (nanozooplankton-N: crosses; microzooplankton-M: open squares; copepods-C: open triangles); SC: *Scrippsiella trochiodea*, DC: *Dictyocha speculum*, TL: *Teleaulax amphioxeia*, CHS: *Chaetoceros similis*, PC: picophytoplankton.

**Figure 5:**
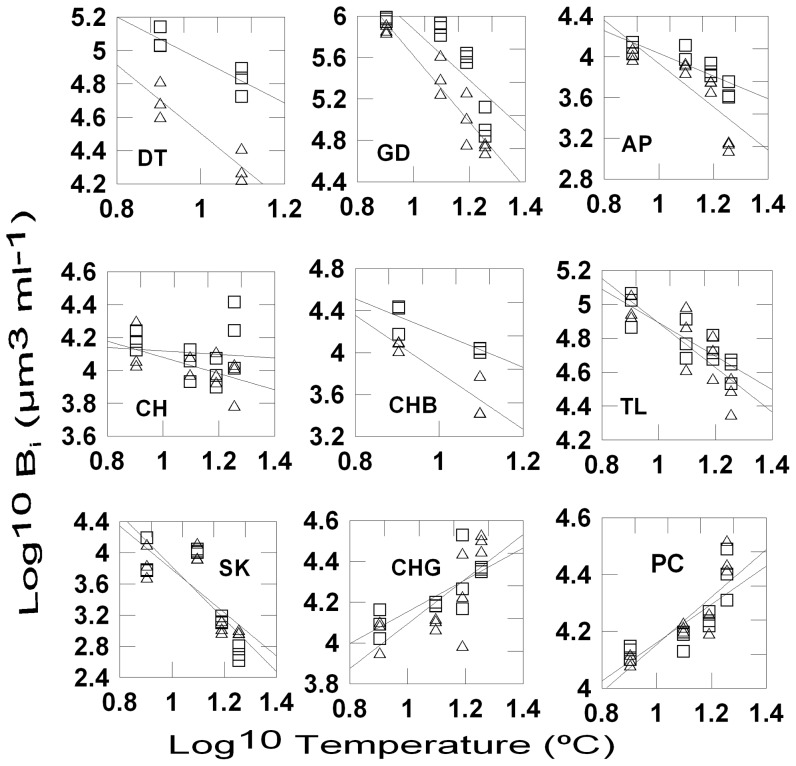
Temperature and grazing effects on the biomass of individual phytoplankton species, experiment 2. Regressions species specific biomass (log^10^ transformed, µm^3^ml^−1^) on temperature (log^10^ transformed, °C) and grazing regimes (microzooplankton-M: open squares; copepods-C: open triangles); DT: *Ditylum brightwellii*, GD: *Guinaridia delicatula*, AP: *Amphidinium* sp., CH: *Chattonella* sp., CHB: *Chaetoceros brevis*, TL: *Teleaulax amphioxeia*, SK: *Skeletonema* cf. *costatum*, CHL: *Chaetoceros gracilis*; PC: picophytoplankton.

In experiment 1, the biomass of *D. speculum, S. trochoidea, T. amphioxeia*, and *Csimilis* showed a significant negative response to temperature, while picophytoplankton showed a positive response (Table 6 and 7, Fig. 4). In experiment 2, a significant negative response to temperature was found in *G. delicatula, A.* sp., *T. amphioxeia, C. brevis*, *D.brightwelii*, and *S.* cf. *costatum*. No significant temperature effect was found in *Chattonella*. The biomass of *C. gracilis* and picophytoplankton increased with temperature (Table 6 and 7, Fig. 5). Grazing treatments had a significant effect on all species in experiment 1, except for picophytoplankton. In all significant cases, biomass decreased with increasing grazer size. The interaction term between temperature and grazer treatment was significant in all cases. In experiment 2, the biomass of *G. delicatula*, *Chattonella* sp., *A.* sp., *C*. *brevis*, and *D. brightwelii* was significantly lower in the C-treatments than in the M-treatments. The biomass of *C. gracilis, T. amphioxeia, S.* cf. *costatum*, and picophytoplankton showed no response to grazing treatment. A significant interaction term between temperature and grazing was only found in *G. delicatula, A.* sp. and *D. brightwellii*.

**Table 6:** Two-factor ANOVA of temperature and grazing effects on biomass of species.

Species	p-temperature	p-grazing	p-interaction	R^2^
**Experiment 1**				
*S. trochoidea*	<0.001	<0.001	0.07	0.79
*D. speculum*	<0.001	<0.001	0.003	0.83
*T. amphioxeia*	<0.001	<0.001	0.003	0.83
*C. simils*	<0.001	<0.001	0.004	0.77
Picophytoplankton	<0.001	0.53	0.06	0.81
**Experiment 2**				
*D. brightwellii*	<0.001	<0.001	0.007	0.89
*G. delicatula*	<0.001	<0.001	<0.001	0.85
*A*. sp.	<0.001	<0.001	0.04	0.82
*Chattonella* sp.	0.07	0.04	0.21	0.34
*C. brevis*	0.004	0.002	0.22	0.83
*T. amphioxeia*	<0.001	0.65	0.73	0.72
*S*. cf. *costatum*	<0.001	0.39	0.5	0.81
*C. gracilis*	<0.001	0.59	0.27	0.58
Picophytoplankton	<0.001	0.53	0.25	0.87

**Table 7:** Regressions (model: y = ax+b) of log^10^ species biomass (µm^3^ml^−1^) on log^10^ temperature (°C) for the different species and grazing regimes.

Species	Grazing	a	b	p	R^2^
**Experiment 1**					
*S. trochoidea*	N	−1.7394	6.4283	0.0003	0.76
	M	−2.5319	6.8926	0.0002	0.84
	C	−2.6512	6.8767	0.0001	0.73
*D. speculum*	N	−0.8629	5.9224	0.027	0.52
	M	−1.1799	6.0683	0.0001	0.80
	C	−1.8966	6.3697	0.00005	0.87
*T. amphioxeia*	N	−0.5537	4.6458	0.006;	0.69
	M	−1.0141	4.841	0.00009;	0.82
	C	−1.7437	5.252	0.0003	0.78
*C. similis*	N	−0.6796	5.2569	0.01	0.73
	M	−1.0356	5.4372	0.03	0.61
	C	−2.0538	5.9669	0.001	0.84
Picophytoplankton	N	+1.6856	2.7581	0.00005	0.81
	M	+1.2065	3.1659	0.001	0.71
	C	+1.2262	3.1846	0.00002	0.86
**Experiment 2**					
*D. brightwellii*	M	−1.2890	6.2320	0.0157	0.80
	C	−2.3933	6.8632	0.0020	0.88
*G. delicatula*	M	−2.4671	8.3421	0.0014	0.65
	C	−3.2014	8.8194	0.00004;	0.89
*A. sp.*	M	−1.1123	5.1461	0.0006	0.70
	C	−2.0581	5.9788	0.0017	0.64
*Chattonella* sp.	M	−0.1064	4.2246	0.7582;	0.0099
	C	−0.4929	4.5723	0.0562	0.32
*C. brevis*	M	−1.6340	5.8205	0.0217	0.76
	C	−2.7162	6.5278	0.0119	0.82
*T. amphioxeia*	M	−0.9858	5.8770	0.0004	0.72
	C	−1.1083	5.9999	0.0004	0.62
*S.* cf. *costatum*	M	−3.3654	7.1868	0.0017	0.64
	C	−2.7763	6.5633	0.0030	0.60
*C. gracilis*	M	+0.7815	3.3717	0.0032	0.59
	C	+1.0952	2.9985	0.0075	0.52
Picophytoplankton	M	+0.6751	+3.4856	0.0017	0.64
	C	+0.8364	+3.3181	0.0008	0.69

## Discussion

### Hypothesis 1

For the majority of species, the predicted decrease in cell size with warming was confirmed. Exceptions where the small diatom *C. gracilis* (experiment 2), the raphidophyte *Chattonella* sp. (experiment 2) and picophytoplankton (both experiments). However, the latter case is not as clear cut, because picophytoplankton is an aggregate category comprising an unknown number of species. Therefore, any size change of this category can also be a consequence of species shifts. The slopes of the size – temperature regressions had a mean value of −0.60 (±0.46 SD) which corresponds to a ca. 4-fold decrease at a one order of magnitude increase in temperature. This is a much stronger effect than the average 2.5% shrinkage per °C reported from meta-analysis of experiments with clonal cultures from a wide array of auto- and heterotrophic protists [26]. At present, we can only offer a tentative explanation for this discrepancy. Contrary to the experiments reported in [26] we did not use clonal i.e. genetically uniform cultures but a natural assemblage which also includes genetic variability within species. Therefore, we also had a selection effect in our experiments, while in clonal cultures size shifts can only result from phenotypic plasticity.

There is a potential caveat for diatoms, because one of the two daughter cells of many diatom species becomes smaller during division. If cell division rates increase with temperature this should lead to an automatic shrinkage of mean size with warming irrespective of other mechanisms. However, faster cell divisions should also lead to a higher biomass accumulation, unless the increased production of cells is removed by increasing losses. While we cannot exclude diatom grazing by copepods, we can exclude grazing by micro- and nanozooplankton for the large celled *D. brightwelii*, *G. delicatula*, *C. brevis* and the chain forming *S*. cf. *costatum* [22], [23]. Protist grazing on the small *C. similis* and *C. gracile* is possible. The latter was the only diatom species whose biomass increased with warming (experiment 2), while the biomass of all other diatom species decreased. We conclude that the diatom division effect did not contribute substantially to the temperature effects on cell size.

The temperature sensitivity of cell size was clearly size dependent. A regression of the slopes *a* from Table 2 on the grand mean of cell sizes of each species (*V_im_*) yielded the following regression (pooled data for both experiments):
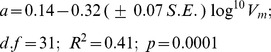



This means, that larger phytoplankton shrink more strongly under warming conditions, an effect which has not yet been reported to the best of our knowledge.

### Hypothesis 2

We found a significant temperature*grazing interaction term in 7 of 14 cases (Table 1). However, these interactions consisted of a change of the negative slope of the size – temperature relationships, but not in a reversal between a negative and a positive dependence. In general, cell sizes were smaller when phytoplankton was subject to larger grazers, a difference which is particularly obvious when comparing the M- and the C-treatments. However, there were some notable exceptions: Picophytoplankton in both experiments, *T. amphioxeia*, *S*. cf. *costatum* and *C. gracilis* in experiment 2.

### Hypothesis 3

Community mean cell size strongly declined with warming. The slopes for this tendency ranged from −1.94 (N-treatments in experiment 1) to ca. −3 (C-treatment in experiment 2), i.e. from a ca. 90-fold to a 1000-fold decrease of community mean cell size at a temperature increase of one order of magnitude. Thus, the interspecific size effect by far exceeds the intraspecific one. While only three species disappeared from the warmer treatments (*C. similis* at 10.5°C in experiment 1, *C. brevis* and *D. brightwellii* at 15.5 and 18.5°C in experiment 2) the relative composition changed to the disadvantage of the large species, which can be seen by a regression analysis of the slopes of the biomass – temperature relationships in Table 6 on cell size:
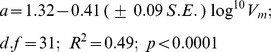



### Hypothesis 4

Community mean cell volume was significantly influenced by grazing and the interaction term temperature*grazing was significant in both experiments. However, while grazing influenced the slope of the temperature response, it did not influence the sign of the relationship. Thus only the weak verion of the hypothesis (4b) was supported while the strong version (4a) was rejected. A switch in sign would have been expected if grazing were the dominant source of size shifts. A higher activity of copepods at higher temperature would have selectively reduced the larger phytoplankton and thereby reduced community mean cell size, while in the absence of copepods a higher activity of protozoans (nano- and microzooplankton) would have selectively removed smaller phytoplankton and thereby increased mean cell volume [27]. It seems that a grazing-independent temperature effect on size effect was strong enough to prevent this reversal of sign. However, as expected, the slope of the community mean cell size – temperature regressions was more negative in the copepod than in the microzooplankton treatments and also more negative in the microzooplankton than in the nanozooplankton treatments of experiment 1.

The shifts in mean cells size are in agreement with the biomass response of the individual species. We found a significant grazer effect on the biomass of phytoplankton species in 8 of 14 cases and significant grazing*temperature interactions in 6 cases. The grazer effect was absent in picophytoplankton in both experiments, and in *T. amphioxeia*, *S.* cf. *costatum* and *C. gracilis* in experiment 2. These were the same species, where also no grazing intraspecific size effect of grazing could be found. Since these were the smallest (experiment 1) or the 4 smallest (experiment 2) species, it seems probable that they were spared from copepod grazing.

The difference between the slopes of the size – temperature regression of the microzooplankton treatments (*a_m_*) and the copepod treatments (*a_c_*) became more negative with cell size:
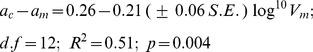



This means, that the increased activity of copepods at higher temperature select smore strongly against larger individuals the bigger the species are. This is in agreement with the known preference of copepods for relatively large phytoplankton [22]. Phytoplankton species exceeding the food niche of copepods in size were lacking in our species pool, but one of the larger species (*S. trochiodea*) showed no copepod effect. *S. trochiodea* is a heavily armored dinoflagellate which is protected from copepod grazing by its cellulose plates [23].

### Alternative explanations and outlook

While our experiments demonstrated an influence of size selective predation on temperature – size relationships, predation cannot be the dominant factor driving temperature - size relationships. Other mechanisms must have been stronger, otherwise the negative temperature size-relationship under protist grazing would not have been possible. Maturation (in our case: cell division) at smaller size as postulated by the TSR [8], [9] can only explain a part of the observed trends. Already the intraspecific effect of most species studied was much stronger than the 2.5% shrinkage per °C found in a meta-analysis of experiments with clonal cultures [26] and shifts between differently sized species had a stronger effect on community mean cell size than size shifts within species.

Our experiments do not support the hypothesis that decreased phytoplankton cell sizes can be explained by intensified nutrient competition at higher temperatures [12], [13], [14], [15], [16]. In stratified oceans and lakes, the increased nutrient stress is caused by increased strength of the vertical stratification and, therefore, decreased upward nutrient supply to the illuminated surface layer. Bottle and mesocosm experiments do not account for the stratification effect on nutrient supply but only for direct temperature effects on nutrient demand. In our experiments, initial availability of nutrients was identical across all treatments and, in agreement with other studies [17], [28], [29], [30], [31], [32] biomass accumulation decreased with warming. This means, that less biomass was built per unit of the limiting nutrient, i.e. biomass specific N-and P-quotas [33], [34], [35], [36] must have been higher under warmer conditions. This conclusion is supported by the N:C ratios in the particulate matter at the end of experiment 2, which we take as a proxy for the biomass specific nitrogen quota. This must have been the quota relevant to assess nutrient limitation, because initial and final dissolved nutrient concentrations indicate a shortage of N relative to P. A two-factor ANOVA shows no significant influence of the grazing regime (p = 0.53) on N:C ratios but a significant effect of temperature (p = 0.0033). A multiple range test (Fisher's LSD) shows two homogenous groups; 8.5 and 12°C with N:C ratios of 0.119±0.010 (S.D.) and 15.5 and 18°C with a N:C ratios of 0.143±0.014 (S.D.). If there is no systematic difference in the biomass specific minimal N-quotas between the warm- and the cold-water communities this would indicate less nutrient stress under warmer conditions. However, smaller phytoplankton tend to have higher biomass specific minimal nutrient quotas, as indicated by an allometry coefficient of 0.56 for the relationship minimal N-quota per cell – cell size [37].

We do not deny the frequently reported effect on nutrient supply on phytoplankton cell sizes which was demonstrated by a recent meta-analysis of size fractionated chlorophyll data from the global ocean [38] but we claim that our results require an explanation different from nutrient supply, grazing and the TSR. Daufresne et al. [10] invoked the metabolic theory of ecology [39], [40] which predicts that at a constant supply rate of the limiting resource biomass should decline with increasing temperature (“energy equivalence rule”) because of increasing metabolic demands per unit biomass. As presented in [10], this explanation is not complete, because there is no logical necessity that the reduction of biomass should be achieved by a reduction of the mean body size instead of a reduction of abundance. However, if warming increases resource demand then it increases resource stress and competition even under constant resource supply. This could lead to a shift towards smaller cell sizes if they are superior competitors [5].
